# Cutaneous metastases as initial presentation of malignancy

**DOI:** 10.1259/bjrcr.20170059

**Published:** 2017-09-13

**Authors:** Supriya Karde, Jayant Sharma, Nagabathula Ramesh, Prashant Bhand, Awanish Shukla

**Affiliations:** ^1^Department of General Medicine, Midlands Regional Hospital, Portlaoise, Co-Laois, Ireland; ^2^Department of Radiology, Midlands Regional Hospital, Portlaoise, Co-Laois, Ireland

## Abstract

We describe a case of 73-year-old female who presented with dry cough, chest pain and light-headedness. On examination, multiple subcutaneous masses were noticed on the chest wall, bilateral breast, anterior abdomen and both arms. Subsequent CT-TAP and CT-brain showed multiple subcutaneous nodules in scalp, neck, anterior chest wall, breast and abdomen. A biopsy taken from breast revealed metastatic malignant melanoma; however there was no evidence of primary cutaneous malignant melanoma. We also describe a case of 72-year-old male who presented with tender mass on his lower back and posterior neck. He was known to have COPD and was a heavy smoker. A CT-TAP showed right lower lung mass with soft tissue masses near lumbar spine and lower cervical spine. While awaiting bronchoscopy, biopsy taken from the mass on lower back showed features of metastatic lung adenocarcinoma. In fact, presence of cutaneous metastasis is heraldsign and indicates advanced malignancy with poor prognosis regardless of type of primary malignancy.

## Background

Generally, all malignancies are associated with cutaneous metastasis and are more common in advanced diseases. It is a well-known fact that the skin is largest organ of the body; however comparatively incidence of cutaneous metastasis is uncommon.^1^ They may represent recurrence of previously treated tumour or can be first manifestation of unrecognized malignancy and therefore is a critical finding. If cutaneous metastasis is the first presentation without a known primary, determining the site of primary tumour may turn out to be a daunting task. It can easily be mistaken for primary tumour and lead to investigations along an unreasonable course. The regional distribution of cutaneous metastasis usually depends on location of the primary disease and mechanism of spread. Certain primary sites may be determined from histopathologic features or immunoprofile of the metastatic deposit.

Presence of cutaneous metastases undeniably indicates disseminated disease and a grim prognosis.^1^

### Case 1

A 73-year-old female presented to the emergency department with 1-week history of dry cough, intermittent light-headedness precipitated by cough, occasional mild chest pain and reduced exercise tolerance. She completed a course of antibiotics 2 weeks ago for respiratory tract infection with partial resolution of symptoms. Background history included asthma, hypertension and hyperthyroidism with total thyroidectomy 3 years ago for large goitre. Regular medications included bisoprolol, lisinopril, levothyroxine and inhalers. Her vitals were otherwise stable. On general examination, multiple subcutaneous nodules over both arms, chest wall and back were noted, they were non-tender, firm, non-pigmented, varying mobility and size. Masses were palpated in bilateral breasts with bilateral axillary and cervical lymphadenopathy. Rest of systemic examination was unremarkable. After further discussion, she mentioned that she only noticed the lumps on her arm a week ago and was not aware of presence of other masses.

**Figure 1.**
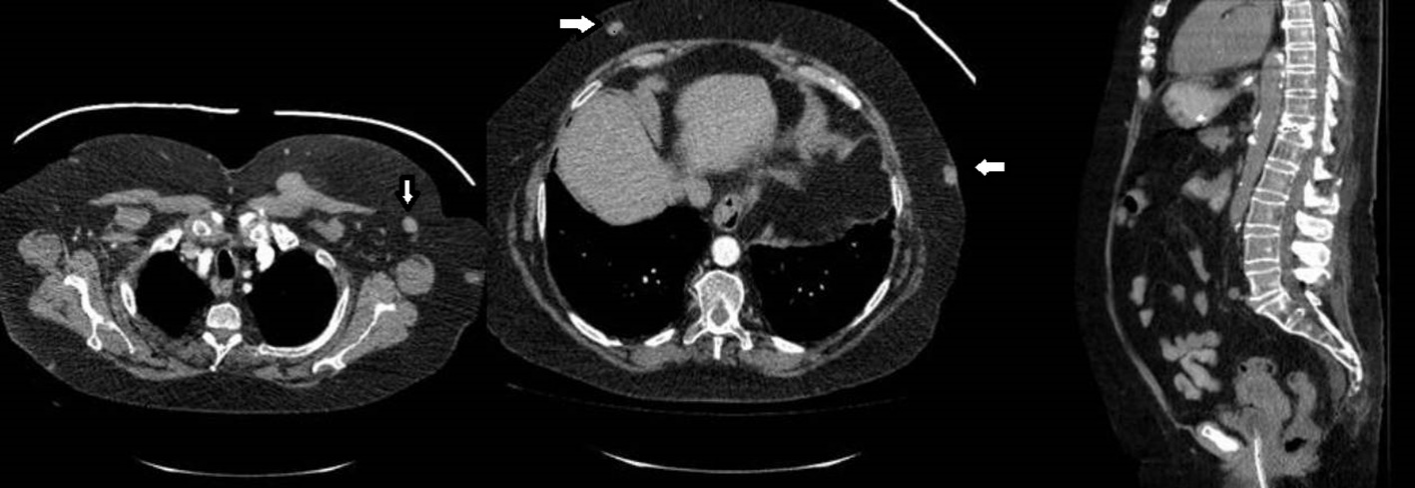
CT-TAP shows multiple heterogeneous soft tissue nodules in neck, chest wall, axilla, anterior abdomen with hilar and mediastinal lymphadenopathy.

Routine lab investigations revealed acute kidney injury with urea of 24.9 mmol l^–1^, creatinine 279 mmol l^–1^, potassium 6.5 mmol l^–1^ and haemoglobin of 10 g dl^–1^. She was immediately commenced on treatment for acute renal impairment and hyperkalaemia. Chest X-ray did not show any abnormality. CT of thorax, abdomen and pelvis was arranged once the renal function normalized which revealed multiple heterogeneous soft tissue nodules in neck, anterior chest wall on the left, axillary and inguinal regions, anterior abdomen with hilar and mediastinal lymphadenopathy; probable lesion in tail of pancreas and cortex of right kidney was described (Figure 1).

**Figure 2.**
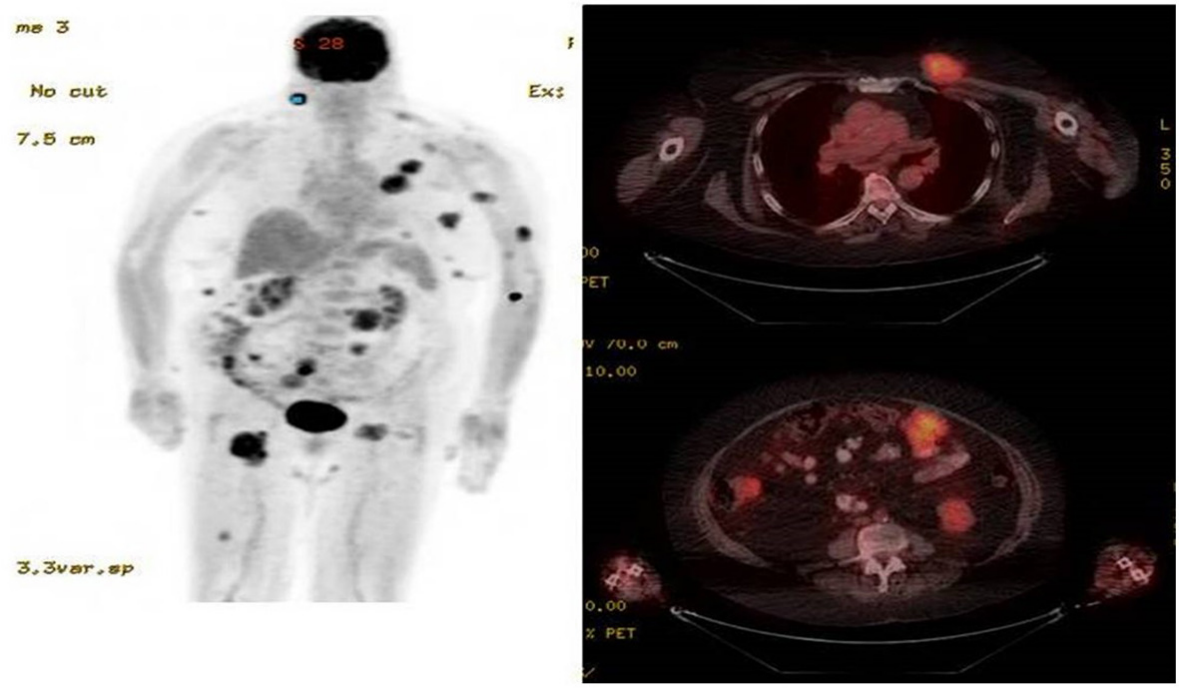
PET scan shows diffuse soft tissue nodules, peritoneal masses and inguinal lymphadenopathy.

Thyroid gland appeared to be normal and no skeletal lesion were seen. Subsequent CT-brain showed heterogeneous nodules in parotid glands bilaterally with multiple soft tissue nodules on the scalp. Possibility of metastatic lesions secondary to breast cancer was considered. PET scan showed diffuse metastatic disease including diffuse soft tissue nodules, peritoneal masses, inguinal lymphadenopathy and intramuscular deposits (Figure 2).

She was then referred to breast clinic where a biopsy from the breast mass and axillary lymph node was taken. The biopsy results showed fatty tissue infiltrated by poorly differentiated malignant neoplasm with epithelioid and plasmacytoid morphology, with some nucleoli and necrosis. The tumour cells were positive for melanocytic markers including S100 and Melan A. HMB45 was negative and so were other epithelial (pan cytokeratin MNF-116, AE1/AE3, CK5/6, p63) and lymphoid (CD45, CD20) markers. The morphology and immunoprofile was consistent with metastatic malignant melanoma. The sample was tested for genetic mutation including BRAF V600E, NRAS, KIT and NTRK1/2/3 fusion of which BRAF V600E mutation was detected. However, the site of primary cutaneous melanoma could not be found.

She was referred to oncology services and commenced on dabrafenib and trametinib. Unfortunately, patient passed away within 6 months of initial diagnosis.

### Case 2

A 72-year-old male presented to emergency department with tender mass on lower back and posterior aspect of neck. Apparently, he had noticed it on his lower back 6 months ago and had grown in size with another similar swelling appearing on his neck, also they were increasingly painful and sore to touch. He had a history of ongoing sore throat for 3 years for which he was extensively investigated by ENT services. His CT neck and mediastinum did not show any abnormalities at that time. This was followed by laryngoscopy which showed a white patch on the right vocal cord, biopsy of which showed non-specific chronic inflammatory changes and cultures grew candida. Background history included COPD; he was heavy current smoker and smoked about 60 cigarettes per day. His medications included salmeterol/fluticasone propionate and ipratropium bromide inhalers. Vitals were within normal range. On examination, 4 cm nodular, firm, tender, immobile mass on lower back and 3 cm mass on posterior aspect of lower cervical spine were noted.

**Figure 3.**
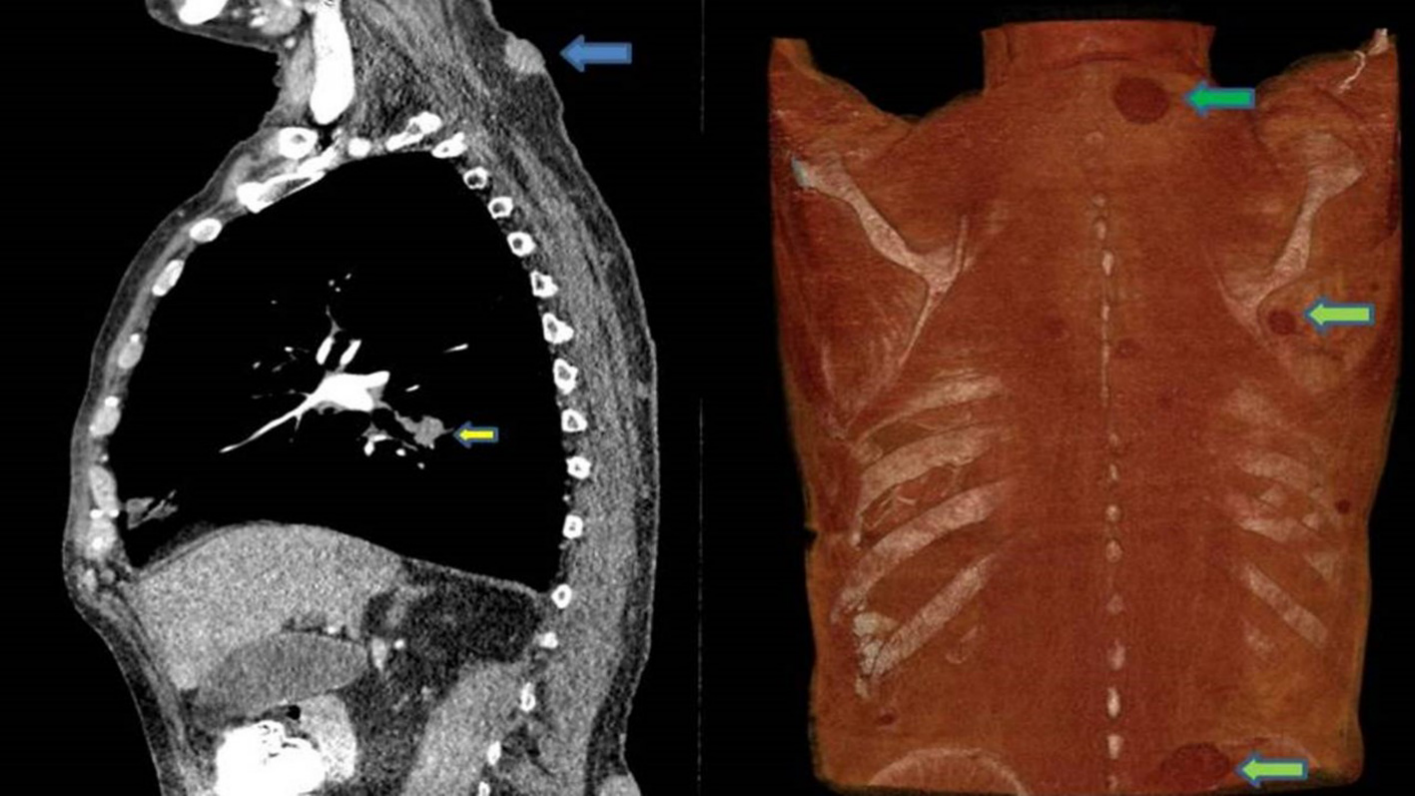
CT-TAP shows 2 x 2 cm mass in right lung lower lobe, 3 x 3cm soft tissue mass at C6-C7 level posteriorly and 4 x 2 cm mass on lumbar region.

His laboratory investigation including renal and liver function, full blood count and coagulation profile were normal. A CT of thorax, abdomen and pelvis was arranged which showed 2 × 2 cm mass in right lung lower lobe likely malignant with no hilar or mediastinal lymphadenopathy, also 3 × 3 cm soft tissue mass at C6-C7 level posteriorly and 4 × 2 cm mass was noted on lumbar region with multiple prominent inguinal lymph nodes (Figure 3).

A referral for bronchoscopy was sent and biopsy of the nodule was planned. While awaiting bronchoscopy appointment, biopsy was taken from mass in lower back and send for histopathology. Histology showed poorly differentiated tumour cells in cohesive groups and sheets with moderate pleomorphism and eosinophilic cytoplasm; immunohistochemistry was positive for AE1/3, cytokeratin 7 and carcinoembryonic antigen antibody stain and periodic-acid schiff. Weak nuclear positivity for TTF-1 was also seen. Overall features were consistent with poorly differentiated adenocarcinoma likely of lung origin.

He was then referred to oncology services and unfortunately passed away within 5 months of diagnosis.

## Discussion

Cutaneous metastasis is an uncommon occurrence though essentially all malignancies have potential to disseminate to skin.^2^ Certain type of malignancies has higher predisposition for cutaneous metastasis compared to others. In general, the incidence of cutaneous metastasis in patients with malignancy is approximately 2 to 10%.^3^ In the clinical setting of a known primary tumour, skin metastasis often occurs with accompanying widespread visceral metastatic disease.^4^ The most common sites of primary malignancies associated with cutaneous metastasis vary according to gender and age. For instance, in women most common primary site of malignancy is breast comprising for 70% of all cutaneous metastases, followed by colon or rectal (10%), melanoma (5%), ovaries (4%) and lungs (4%).^5^ On the other hand, in men the most common primary site associated with metastasis to skin is lung accounting for 25%, followed by colon (20%).^5^ It has been observed that cutaneous metastasis tends to develop within months to years of initial diagnosis for most tumour types, although in 7% of cases they may emerge even after a lapse of 5 years.^4^ Melanoma and breast carcinoma are the most common tumour types to present with delayed cutaneous metastases. In 0.8 to 10% cases it can be first sign of underlying undiagnosed malignant disease while in others it may indicate recurrence in previously treated disease.^4^ The incidence may be even higher in children where surprisingly, it may be first clinical manifestation in 84% of cases. Alarmingly, up to 50% of malignant cutaneous deposits in children are metastatic.^4^

Malignancies may spread to skin either by direct spread from adjacent non-cutaneous structure, lymphatic or haematogenous spread, and infrequently by implantation following surgical or diagnostic procedure.^4^ The regional distribution of cutaneous metastasis although variable, usually depends on the location of the primary disease and mechanism of spread. Carcinoma of the kidney and lung tend to invade vein and often present as metastasis to skin site distant from primary tumour.^5^ Cancers that invade lymphatics, for instance carcinoma of the breast and squamous cell carcinoma of the oral cavity, appear late in the course of the disease and may invade skin overlying the primary tumour.^5^ The abdominal wall is the most common site for tumours presenting as metastatic disease.^5^ Scalp metastases in females should raise a suspicion of possible breast cancer and in males of lung malignancy.^5^

The most common representation of cutaneous metastasis is an aggregate of discrete, firm, non-tender, skin-coloured nodules that appear suddenly, grow rapidly, attain a certain size and may remain stationary thereafter.^3^ These lesions may be mistaken for cysts or benign fibrous tumours and at times the clinical picture may mimic vascular tumours.^3^ In such circumstances, accurate diagnosis may be difficult and involve exasperating line of investigations. Whole body PET scan may reveal unsuspected primary sites of disease and is a better than CT in demonstrating cutaneous metastases.^6^ Metastatic skin tumours can be distinguished from primary cutaneous tumour by histopathologic studies, molecular staining and immunoprofile.^7^ The chemokine receptors and ligand binding pairs CCL27/CCR10 has been demonstrated to be involved in cutaneous metastatic melanoma and may mediate melanoma survival in the skin.^1,8^

The treatment is clearly governed by the type of primary malignancy. Unfortunately, the prognosis of cutaneous metastatic disease is poor regardless of type of primary neoplasm.

## Learning points

1. It is undeniable that such skin signs may seem harmless to inexperienced eyes especially in patients with unrelated symptoms and disease.
2. Indeed, such unexplained skin findings should invite speculation of underlying sinister disease and prompt immediate investigation.
3. In case of known malignancy, cutaneous metastasis is a portentous sign and may potentially change disease staging and therapy.

## Consent

Written informed consent for the case to be published (including images, case history and data) was obtained from the patient(s) for publication of this case report, including accompanying images.
